# Does Day-to-Day Variability in Stool Consistency Link to the Fecal Microbiota Composition?

**DOI:** 10.3389/fcimb.2021.639667

**Published:** 2021-07-20

**Authors:** Lisa Vork, John Penders, Jonna Jalanka, Svetlana Bojic, Sander M. J. van Kuijk, Anne Salonen, Willem M. de Vos, Mirjana Rajilic-Stojanovic, Zsa Zsa R. M. Weerts, Ad A. M. Masclee, Marta Pozuelo, Chaysavanh Manichanh, Daisy M. A. E. Jonkers

**Affiliations:** ^1^Division of Gastroenterology-Hepatology, Department of Internal Medicine, NUTRIM School of Nutrition and Translational Research in Metabolism, Maastricht University Medical Center+, Maastricht, Netherlands; ^2^Department of Medical Microbiology, NUTRIM School of Nutrition and Translational Research in Metabolism, Maastricht University Medical Center+, Maastricht, Netherlands; ^3^Human Microbiome Research Program, Faculty of Medicine, University of Helsinki, Helsinki, Finland; ^4^Department of Biochemical Engineering and Biotechnology, Faculty of Technology and Metallurgy, University of Belgrade, Belgrade, Serbia; ^5^Clinical Epidemiology and Medical Technology Assessment, Maastricht University Medical Center+, Maastricht, Netherlands; ^6^Laboratory of Microbiology, Wageningen University, Wageningen, Netherlands; ^7^Digestive System Research Unit, University Hospital Vall d’Hebron, Barcelona, Spain; ^8^Centro de Investigación Biomédica en Red Enfermedades Hepáticas y Digestivas (CIBERehd), Instituto de Salud Carlos III, Madrid, Spain

**Keywords:** fecal microbiota, intestinal microbiome, stool consistency, irritable bowel syndrome, adult

## Abstract

**Introduction:**

Stool consistency has been associated with fecal microbial composition. Stool consistency often varies over time, in subjects with and without gastrointestinal disorders, raising the question whether variability in the microbial composition should be considered in microbiota studies. We evaluated within-subject day-to-day variability in stool consistency and the association with the fecal microbiota in irritable bowel syndrome (IBS) and healthy subjects, over seven days.

**Methods:**

Twelve IBS patients and 12 healthy subjects collected fecal samples during seven consecutive days. Stool consistency was determined by the patient-reported Bristol Stool Scale (BSS) and fecal dry weight percentage. 16S rRNA V4 gene sequencing was performed and microbial richness (alpha diversity; Chao1 index, observed number of species, effective Shannon index) and microbial community structure (beta diversity; Bray-Curtis distance, generalized UniFrac, and taxa abundance on family level) were determined.

**Results:**

Linear mixed-effects models showed significant associations between stool consistency and microbial richness, but no time effect. This implies that between-subject but not within-subject variation in microbiota over time can partially be explained by variation in stool consistency. Redundancy analysis showed a significant association between stool consistency and microbial community structure, but additional linear mixed-effects models did not demonstrate a time effect on this.

**Conclusion:**

This study supports an association between stool consistency and fecal microbiota, but no effect of day-to-day fluctuations in stool consistency within seven days. This consolidates the importance of considering stool consistency in gut microbiota research, though confirms the validity of single fecal sampling to represent an individual’s microbiota at a given time point. NCT00775060.

## Introduction

Over the past decades, the gut microbiota has been studied extensively in the context of gastrointestinal (GI) function in health and disease. Stool consistency measured with the Bristol Stool Scale (BSS) or fecal moisture has been associated with gut microbiota diversity and composition (Tigchelaar et al., 2016; Vandeputte et al., 2016), and has been identified as important covariate of the microbiota composition in population-based studies (Falony et al., 2016; Vandeputte et al., 2017). This indicates the importance of considering stool consistency as a potential confounding factor in both cross-sectional and longitudinal intestinal microbiota analyses. However, stool consistency can fluctuate over time (Matto et al., 2005), possibly associated with temporal fluctuations in the microbiota composition. This raises the question whether a single fecal sample is representative for a subject’s microbial profile at a given time point and whether within-subject day-to-day variability should be considered in microbiota studies, by collecting repeated fecal samples.

While stool consistency can fluctuate from day to day in healthy subjects, fluctuations are often more pronounced in subjects with irritable bowel syndrome (IBS), a GI disorder characterized by abdominal pain and altered bowel habits. According to the Rome criteria, IBS is typically divided into four subtypes, often defined by BSS: diarrhea predominant (IBS-D), constipation predominant (IBS-C), a combination of both (IBS-M; mixed type), or unspecified in which both diarrhea and constipation are not predominantly present (IBS-U) (Longstreth et al., 2006). Multiple studies have shown changes in gut microbiota composition as well as functionality in IBS compared to healthy subjects (Salonen et al., 2010; Rajilic-Stojanovic et al., 2011; Jeffery et al., 2012). Some results point towards differences in microbiota between subgroups of IBS subjects (Malinen et al., 2005; Kassinen et al., 2007; Jeffery et al., 2012), though findings on specific bacterial taxa are not consistent. Additionally, in cross-sectional studies changes in microbial composition have been correlated to IBS symptom scores (Malinen et al., 2010; Rajilic-Stojanovic et al., 2011; Jeffery et al., 2012), which also generally vary over time.

So far, a few studies have focused on temporal (in)stability of the fecal microbiota and found a more unstable microbial composition in IBS patients compared to healthy volunteers over a period of months (Matto et al., 2005; Lyra et al., 2009; Durban et al., 2013). However, none have considered possible day-to-day variation and its association with stool consistency and symptoms within subjects over time. Since both stool consistency and GI symptoms can fluctuate from day to day, especially in IBS populations, a key question is whether a single fecal sample at one time point suffices or whether repeated sampling over a period of time is needed in order to take into account day-to-day variability in microbial composition.

Therefore, we aimed to evaluate within-subject day-to-day variability in stool consistency and gastrointestinal symptoms, and the association with the fecal microbiota composition in IBS and healthy subjects, over a seven-day course.

## Methods

### Study Design and Participants

This study was embedded in the Maastricht IBS Cohort. The study protocol has been approved by the Maastricht University Medical Center+ (MUMC+) Committee of Ethics (METC 08-2-066) and was executed according to the revised Declaration of Helsinki (64^th^ WMA General Assembly, Brazil 2013). The study has been registered in the US National Library of Medicine (http://www.clinicaltrials.gov, NCT00775060).

Between January 2015 and March 2016, IBS patients aged 18-75 years were recruited at the outpatient department of Gastroenterology-Hepatology of MUMC+. All subjects fulfilled the Rome III criteria and were assigned to IBS subtypes based on predominant bowel habits (Drossman, 2006). A history of abdominal surgery, except for uncomplicated appendectomy, cholecystectomy, or hysterectomy, was reason for exclusion. Additional investigations to exclude organic disease were performed if deemed necessary by a gastroenterologist.

Age- and sex-matched healthy controls (HC) were recruited *via* public advertising and recruitment websites. A brief medical history was taken by a trained research physician to exclude the presence of any GI disorders or current GI symptoms. All participants gave written informed consent prior to inclusion.

### Bio Samples and Symptom Scores

A seven-day symptom diary was used to record daily symptom scores and bowel habits using BSS [Lewis and Heaton, 1997; U.S. Department of Health and Human Services FaDA and Center for Drug Evaluation and Research (CDER), 2012]. Symptoms (*i.e.* abdominal pain, bloating, fecal urgency, diarrhea, and constipation) were scored at the end of the day on an 11-point numeric rating scale [*i.e.* 0 (none) to 10 (severe) [U.S. Department of Health and Human Services FaDA and Center for Drug Evaluation and Research (CDER), 2012] and BSS for stool consistency was reported for every bowel movement. Additionally, medication use was reported every day and participants were instructed to maintain their habitual dietary habits during the study period.

The first fecal samples of each day, and one additional sample when subjects reported diarrhea, were collected. Subjects stored the samples at -20°C at home directly after collection. Following the seven-day study period, all samples were transported to MUMC+ on dry ice and stored at -80°C.

In addition to the BSS, the dry weight content of each fecal sample was determined, as a more objective measure of stool consistency. Therefore, an aliquot of 0.5 g (*wet weight*) was dried at 60°C in a vacuum dryer for 5 hours. Directly after drying, the samples were weighed again (*dry weight*). The percentage of dry weight was calculated as: [dry weight(g)/wet weight(g)] * 100.

### Fecal Microbiota Analysis

Fecal microbiota profiling was achieved by next-generation sequencing of 16S rRNA V4-region gene amplicons. Detailed information on DNA extraction, sequencing, and data analysis can be found under **Supporting Information – Supplementary Methods**.

### Statistical Analysis

All statistical analyses were performed using QIIME version 1.9.1 and R version 3.4.2. Categorical patient characteristics are presented as proportions and differences between groups were tested using χ^2^ or Fisher’s exact test. Continuous characteristics are presented as mean and standard deviation (SD) or median with interquartile range in case of skewness. Differences between groups were tested using the independent t-test or the Mann-Whitney U test, depending on the normality of the distribution.

Alpha diversity data are expressed as Chao1 index, observed species, and effective Shannon index (exp[Shannon index]). To evaluate the within-subject variability in alpha diversity over time, inter-item (Pearson) correlations between the consecutively collected fecal samples, as well as intra-class correlations (ICC), were calculated for IBS and healthy subjects separately. Data from subjects that collected at least five consecutive samples were included in these analyses. The Bray-Curtis distance and generalized UniFrac (*i.e.* beta diversity) were used to quantify the (dis)similarity in microbial community structure between samples. Non-parametric tests were used for taxonomical abundance data. Principal coordinates analyses (PCoA) were performed on beta diversity indices to evaluate possible clustering of the microbial community structure. A Mann Whitney-U test was used to compare the average within-subject diversity distance between IBS and healthy subjects.

To evaluate the correlation between stool consistency and the microbiota, a constrained redundancy analysis (RDA) was carried out, using routines from R package “vegan”. The zeros from the count data (summarized on family level) were imputed using R package “zCompositions” and data were clr (centered log ratio) transformed. In addition, two-level mixed-effects linear regression models (level 1: consecutive stool samples; level 2: subjects) were used to examine the association between stool consistency and microbiota using all longitudinal measurements. Separate models were used for different measures of microbial diversity and composition, with alpha diversity indices and clr transformed taxonomical abundance data (family level), respectively, as the dependent variables. The two-way interaction term “stool consistency*time” and stool consistency were used as independent variables and a random intercept was chosen to correct for clustering of multiple measurements within each participant. A p-value of ≤0.05 was considered statistically significant.

In all analyses, stool consistency was primarily based on fecal dry weight percentage, and additional analyses were performed using BSS.

## Results

### Study Population

Twelve IBS patients and 12 healthy subjects were included. Demographics are shown in **Table 1**. In total, 71 and 78 samples were available for the IBS and healthy group, respectively.

**Table 1:** Demographic characteristics.

	Healthy (n=12)	IBS (n=12)
**Female sex**, n (%)	10 (83.3)	10 (83.3)
**Age** in years, median [IQR]	36.78 [30.09 – 47.19]	45.0 [35.37 – 48.56]
**BMI** in kg/m2, median [IQR]	21.99 [21.80 – 23.25]	23.65 [22.65 – 24.57]
**Current smoking**, n (%)	0 (0)	4 (33.3)
**Current alcohol use** (<15 units/week), n (%)	6 (50)	7 (58.3)
**Number of fecal samples^1^**, n (%)		
1	-	-
2	-	1 (8.3)
3	-	-
4	1 (8.3)	-
5	-	4 (33.3)
6	4 (33.3)	2 (16.7)
7	6 (50.0)	3 (25.0)
8	1 (8.3)	2 (16.7)
**GSRS**, median [IQR]		
Abdominal Pain	1.67 [1.08-2.25]	3.33 [2.00-4.67]^$^
Regurgitation Syndrome	1.00 [1.00-1.00]	2.50 [1.00-3.50]^#^
Diarrhea Syndrome	1.00 [1.00-1.33]	3.33 [1.00-1.33]^$^
Indigestion Syndrome	1.75 [1.50-2.50]	4.75 [3.75-5.13]^$^
Constipation Syndrome	1.67 [1.00-1.92]	3.33 [2.33-4.67]^$^
**Medication use**, n (%)		
PPI	-	3 (25)
NSAID	-	-
Prokinetic	-	-
Spasmolytic	-	2 (16.7)
Laxative	-	1 (8.3)
Antidiarrheal	-	-
Antibiotic	-	-
Probiotic	-	-
Prebiotic	-	-
Other	7 (58.3)	7 (58.3)

Differences tested using Mann-Whitney U test for continuous data and χ^2^- or Fisher’s exact test for categorical data. Significances are shown for IBS versus healthy. *p < 0.05; ^#^p < 0.01; ^$^p < 0.001.

^1^Less than one fecal sample per day was collected in case of absence of bowel movement; more than one fecal sample per day was collected in case of diarrhea.

The day-to-day variability in stool consistency, measured by fecal dry weight percentage, was found to be high in the IBS group (ICC: 0.223) and moderate (ICC: 0.622) in the healthy population. BSS scores showed high day-to-day variability in both groups (ICC for IBS: 0.397; ICC for HC: 0.276). This variability in stool consistency is illustrated in **Supplementary Figure 1**.

### Day-to-Day Variability of the Microbiota

The predominant phyla in both healthy and IBS subjects were Firmicutes (average relative abundance of 79.5% and 81.9%, respectively), followed by Bacteroidetes, Actinobacteria, Verrucomicrobia, and Proteobacteria (**Supplementary Figure 2**). The relative abundances of these five predominant phylae are shown in **Supplementary Figure 3**. While evaluating differences between IBS and healthy subjects is beyond the scope of this study, the abundances are shown for both groups separately for better visibility.

For both IBS and healthy subjects, microbial richness showed high correlations between subsequent samples, demonstrating low within-subject variability from day to day. Inter-item correlations between different samples were all above 0.800 and a high degree of agreement in microbial richness was found between the different samples of one subject (single measure ICCs all above 0.893). Inter-item matrices and ICCs for Chao1 index are shown in **Table 2**. Similar results were found for observed species, and correlations were moderate for effective Shannon index (**Supplementary Tables 1A**, **B**).

**Table 2:** Inter-item (Pearson) correlations between Chao1-index of consecutive samples, for healthy subjects and IBS patients.

Chao1 index	HEALTHY SUBJECTS				IBS PATIENTS			
	Intraclass Correlations Coefficient = 0.940				Intraclass Correlations Coefficient = 0.893			
	Sample 1	Sample 2	Sample 3	Sample 4	Sample 1	Sample 2	Sample 3	Sample 4
**Sample 2**	0.967				0.923			
**Sample 3**	0.866	0.922			0.870	0.882		
**Sample 4**	0.972	0.954	0.907		0.853	0.910	0.908	
**Sample 5**	0.946	0.989	0.936	0.925	0.916	0.895	0.916	0.915

**Figure 1:**
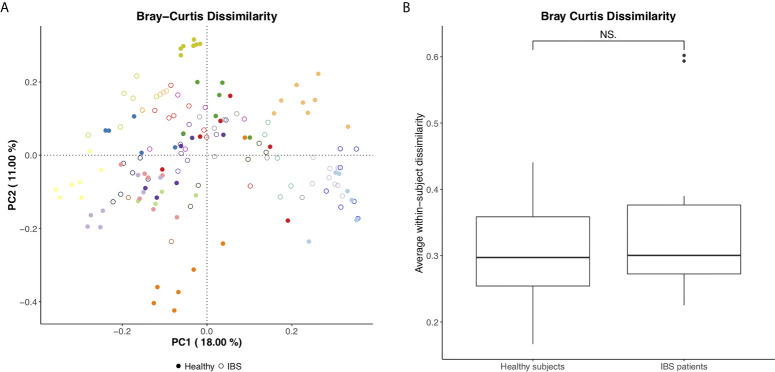
**(A)** Bray Curtis PCoA plot per individual (different colors) and for healthy subjects *vs.* IBS patients (figure annotations). **(B)** Average within-subject beta diversity (Bray Curtis Dissimilarity) for healthy subjects *vs.* IBS patients, significance tested using Mann Whitney U test, NS, not significant (p=0.71).

The microbial community structure clustered per individual, suggesting that the dissimilarity in microbiota between consecutive samples is larger between than within subjects, while no clear separation between IBS and healthy subjects was demonstrated (**Figure 1A**). Moreover, no significant difference was found in the average within-subject beta diversity between IBS and healthy subjects, indicating that the day-to-day variation in overall microbial community structure is similar between IBS and healthy subjects (**Figure 1B**). Comparable results were found for the generalized UniFrac as measure of beta diversity (**Supplementary Figures 4A, B**). In addition, in order to evaluate whether the dissimilarity in microbiota between different samples (*i.e.* within individual subjects) becomes larger over time, we illustrate the Bray Curtis Dissimilarity for each day (*i.e.* day 2 – 7) compared to day 1, separately for each subject in **Supplementary Figure 5A** (IBS patients) and **Supplementary Figure 5B** (healthy subjects). No apparent increase in beta diversity over the seven days is shown, indicating a rather stable microbial community structure per individual during the study period.

### Association Between Microbiota and Stool Consistency

A linear mixed-effects model with the Chao1 index as dependent variable demonstrated no significant effect of the two-way interaction “fecal dry weight*time” on microbial richness (B: 0.030, SE: 0.072, p=0.676), indicating that the association between stool consistency and microbial richness was not different between subsequent samples. After removal of this term from the model, stool consistency was found to be a significant predictor of microbial richness (B: 1.231, SE: 0.200, p<0.001) (**Table 3**), showing an overall significant association between stool consistency and the microbiota, but independent of time. Subsequent linear mixed-effects models with observed species and the effective Shannon index as dependent variables, showed similar results (**Supplementary Tables 2A**, **B**). Models using BSS instead of fecal dry weight as a measure of stool consistency also showed similar results (**Table 3**, **Supplementary Tables 2A**, **B**).

**Table 3:** Results of linear mixed-effects models.

Dependent variable	Predictor	Regression coefficient [95%-CI]	SE	p-value
*Chao1 index*	Fecal dry weight*time	0.030 [-0.113; 0.173]	0.072	0.676
	Fecal dry weight^1^	1.231 [0.835;1.628]	0.200	<0.001
	BSS*time	0.135 [-0.710; 0.980]	0.426	0.752
	BSS^1^	-2.518 [-4.728; -0.308]	1.116	0.026
	Abdominal pain*time	-0.034 [-0.436; 0.368]	0.203	0.869
	Abdominal pain^1^	0.473 [-1.188; 2.135]	0.839	0.574
	Abdominal bloating*time	0.471 [-0.019; 0.962]	0.248	0.059
	Abdominal bloating^1^	0.182 [-1.572; 1.937]	0.886	0.837

Linear mixed-effects models with random intercept, fixed slopes, and scaled identity covariance structure. Regression coefficient indicates the direction and strength of the association between the predictor and dependent variable.

^1^Insignificant interaction terms, respectively, “fecal dry weight*time”, “BSS*time” “abdominal pain*time”, and “abdominal bloating*time” were removed from the models. BSS, Bristol Stool Scale; SE, standard error.

**Figure 2:**
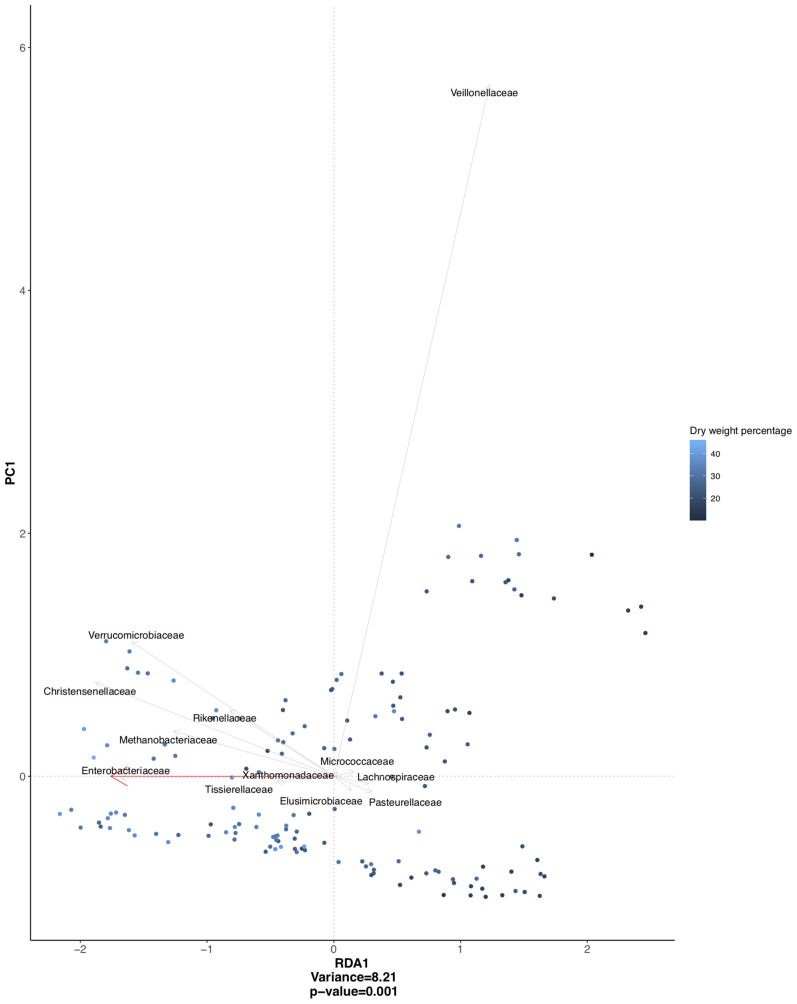
Redundancy analysis plot based on clr transformed abundancies, and constrained on stool consistency (dry weight percentage), with individual variation partialled-out. Significant association between stool consistency and microbial composition (p = 0.001), mainly driven by bacterial families depicted in the figure.

A redundancy analysis, including data from both IBS and healthy subjects, showed a significant association between stool consistency and microbial composition on the family level, mainly driven by *Christensenellaceae, Enterobacteriaceae, Verrucomicrobiaceae, Methanobacteriaceae*, and *Veillonellaceae* (**Figure 2**). To correct for within-subject day-to-day variability in these associations between specific bacterial groups and stool consistency, linear mixed-effects models with the respective bacterial groups as the dependent variable were performed. For *Christensenellaceae, Enterobacteriaceae, Verrucomicrobiaceae*, and *Methanobacteriaceae*, stool consistency was indeed a significant predictor (*i.e.* higher abundance in firmer stools), but not for *Veillonellaceae*.

### Association Between Microbiota and GI Symptoms

**Figure 3:**
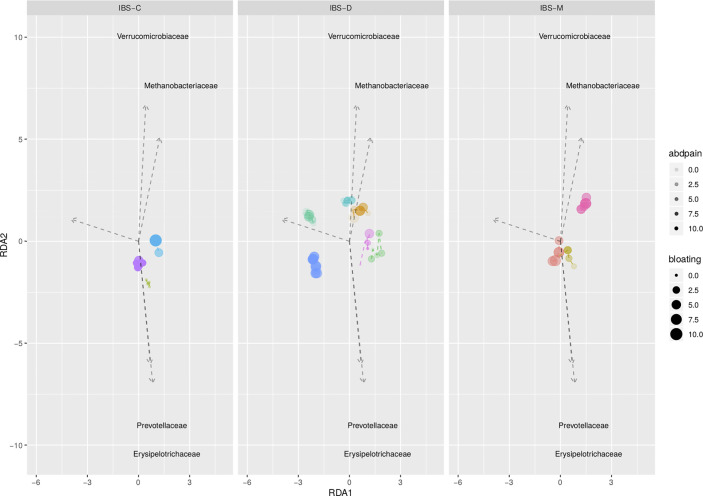
Redundancy analysis plot based on clr transformed abundancies, and constrained on individual, abdominal pain, and abdominal bloating. Each dot represents an individual sample; IBS patients are depicted by different colors. No significant association between both GI symptoms (p = 0.368 for abdominal pain; p = 0.521 for bloating) and microbial composition.

Exploratory analysis showed no significant association between the fecal microbiota (*i.e.* alpha and beta diversity) and abdominal pain and bloating (**Figure 3**), and can be found in **Supporting Information – Supplementary Results**.

## Discussion

Previously described strong correlations between stool consistency and the fecal microbiota pointed towards the importance of stool consistency and/or gut transit time as confounding factors in microbiota analyses (Falony et al., 2016; Vandeputte et al., 2016; Vandeputte et al., 2017). Our study confirms this, though demonstrates that within-subject variation in the fecal microbiota composition between consecutive samples over seven days is limited in both IBS and healthy subjects, even in case of fluctuating stool consistency or GI symptoms.

Stool consistency is known to vary within individuals over time (Matto et al., 2005), suggesting that the fecal microbiota may also change over short time periods. Therefore, this study focused on the microbial stability from day to day and the association with stool consistency. Both IBS patients and healthy subjects were evaluated, since stool consistency is known to show high within-subject variability from day to day in IBS, but variability occurs in healthy subjects as well. We here demonstrate low within-subject day-to-day variability in fecal microbiota over seven consecutive days. Overall variability in the microbial community structure was mainly driven by between-subject variability, with rather small within-subject changes between consecutive samples. Although significant associations between stool consistency and microbial richness and composition were found, no significant effect of time on this association could be demonstrated. Altogether, this indicates that regardless of fluctuations in stool consistency, the variation in fecal microbiota composition within subjects over a one-week period, in both IBS patients and healthy subjects, is rather limited when compared to variations between subjects.

Previous results on temporal stability of the microbial composition are contradictory. Particularly inter-individual differences have been demonstrated, but less is known about within-subject microbial variability (Jalanka-Tuovinen et al., 2011; Rajilic-Stojanovic et al., 2012). Instability in both IBS and healthy subjects has been demonstrated, with possibly a more instable microbiota in IBS patients, assessed over three to six months (Matto et al., 2005; Maukonen et al., 2006). Others found low variability between fecal microbial profiles in healthy subjects with intervals of 14 days to several months. However, even during periods of overall community stability, intra-individual dynamics of select microbial taxa could still be observed (Zoetendal et al., 1998; Zoetendal et al., 2001; Vanhoutte et al., 2004; Jalanka-Tuovinen et al., 2011; Rajilic-Stojanovic et al., 2012; Johnson et al., 2019). According to our results, such within-subject variation in microbial profiles is limited in both IBS and healthy subjects and not related to stool consistency over a seven-day period.

We used fecal dry weight percentage and patient-reported BSS scores as measures of stool consistency. From day to day, moderate-to-high levels of variability in both measures were found in both groups. This confirms that our data should be suitable to detect any day-to-day changes in the microbiota related to variability in stool consistency. Previous findings demonstrated that looser stools, according to BSS, were associated with lower species richness (Vandeputte et al., 2016). Our findings, both using fecal dry weight percentage and the BSS, confirm this association, but in addition indicate that there is no major within-subject shifting in microbial richness and composition from day to day, associated with short-term changes in stool consistency. Also, the previously demonstrated correlation between specific genera abundances and stool consistency was not dependent of time in our study. An increase in abundance of members of the *Methanobacteriaceae* and *Verrucomicrobiaceae*, and the *Enterobacteriaceae* and *Christensenellaceae* families in association with firmer stools has been described (Tigchelaar et al., 2016; Vandeputte et al., 2016; Vandeputte et al., 2017; Jalanka et al., 2019). However, linear mixed-effects models could not confirm an association between *Veillonellaceae* and stool consistency, suggesting that this association is mainly driven by between-subject differences and becomes less important when correcting for within-subject variation. In addition to these previous findings, we could not demonstrate important within-subject shifting in these bacterial groups in relation to fluctuations in stool consistency.

Since previous studies suggested a link between the microbiota and abdominal symptoms in IBS (Malinen et al., 2010; Rajilic-Stojanovic et al., 2011; Jeffery et al., 2012), and these can vary from day to day, we exploratory evaluated this association for abdominal pain and bloating. Our results indicate that there are no daily shifts in the gut microbiota related to daily individual symptom patterns.

Previous studies also showed rapid changes of microbial composition over the course of several days after dietary changes, especially on animal-based diet (David et al., 2014). This demonstrates that the gut microbial community can change rapidly and underlines the importance of considering time when evaluating potential confounders. In the current study, participants were not allowed to introduce major dietary changes, and we do not expect diet to be an important confounder in our results.

This is the first study to examine a short-term within-subject association between stool consistency and the gut microbiota, using repeated fecal sampling over one week. Both IBS and healthy subjects were evaluated in order to capture highly fluctuating as well as more stable day-to-day patterns of stool consistency, but this study was not designed to draw any conclusions on differences in the microbial composition between IBS and healthy subjects. Fecal dry weight percentage was used as an objective measure of stool consistency, which might have an advantage over the commonly used Bristol Stool Scale, since the latter was developed as a surrogate marker of whole-gut transit time and is subject to inter-individual differences in interpretation (Lewis and Heaton, 1997). A more detailed recommendation on the use of fecal dry weight percentage to correct for stool consistency in future (microbiota) studies was recently published elsewhere (Vork et al., 2019). Nevertheless, results were similar for BSS scores and fecal dry weight percentage, confirming that our results are suitable to make comparisons with previous studies. The results on the day-to-day stability of microbial richness were slightly less pronounced for the effective Shannon index when compared to Chao1 index and observed species. The Shannon index is a measure of biodiversity, taking into account the evenness next to the richness, and is therefore more affected by shifts in abundance of microbial taxa. Nevertheless, we could not demonstrate a significant effect of time on the association between stool consistency and microbial richness for any of the three measures.

A sample size of 24 subjects might be relatively small, but the use of repeated measures increases statistical power (Schuster et al., 2020). Furthermore, the performed analyses focus on within-subject rather than on between-subject differences. The female predominance in our study population reflects the sex distribution in IBS, which might question the generalizability to the general population. Previous studies, however, demonstrated no important differences in microbial composition between men and women (Arumugam et al., 2011; Falony et al., 2016).

In conclusion, this study supports an association between stool consistency and the fecal microbiota, but the overall microbial composition was not significantly related to day-to-day fluctuations in stool consistency. This consolidates the importance of considering stool consistency in gut microbiota research, but on the other hand, confirms the validity of the use of single fecal sampling for between-subject comparisons in the context of cross-sectional studies. Likewise, this indicates that in longitudinal studies evaluating within-subject microbial stability over longer periods (*i.e.* months or years) one fecal sample per time point should suffice. Concluding, a single fecal sample appears sufficient to gain a reliable picture of an individual’s microbiota within a short time period, even within subjects with large fluctuations in stool consistency.

## Corporate Authors from the COST Action GENIEUR/BM1106 Microbiome Working Group

Beate Niesler Chair COST-Action GENIEUR.
Department of Human Molecular Genetics, Institute of Human Genetics, Heidelberg University Hospital, Heidelberg, Germany.
nCounter Core Facility, Institute of Human Genetics, Heidelberg University Hospital, Heidelberg, Germany.
Interdisciplinary Center for Neurosciences (IZN), Heidelberg University, Heidelberg, Germany.
Gerard Clarke Department of Psychiatry and Alimentary Pharmabiotic Centre, University College Cork, Cork, Ireland.
Paul Enck Department of Psychosomatic Medicine and Psychotherapy, University Hospital Tuebingen, T̈übingen, Germany
Natasa Golic Laboratory for Molecular Microbiology, Institute of Molecular Genetics and Genetic Engineering, University of Belgrade, Belgrade, Serbia.
Kurt Hanevik Department of Clinical Science, University of Bergen, Bergen, Norway.
Fuad A. Iraqi Department of Clinical Microbiology and Immunology, Sackler Faculty of Medicine, Tel-Aviv University, Tel-Aviv, Israel.
Elena Philippou Department of Life and Health Sciences, University of Nicosia, Cyprus.
Division of Diabetes and Nutritional Sciences, King’s College London, London, UK.
Jeroen Raes Department of Microbiology and Immunology, KU Leuven, Leuven, Belgium.
Robin C. Spiller Nottingham Digestive Diseases Biomedical Research Unit, University of Nottingham, Queens Medical Centre, Nottingham, UK.

## Data Availability Statement

The datasets presented in this study can be found in online repositories. The names of the repository/repositories and accession number(s) can be found below: https://www.ncbi.nlm.nih.gov/, PRJNA682378.

## Ethics Statement

The studies involving human participants were reviewed and approved by Medisch-etische toetsingscommissie azM/UM. The patients/participants provided their written informed consent to participate in this study.

## Author Contributions

Study concept and design: JP, WV, and DJ. Collection study materials: LV and ZW. Data analysis: LV, JJ, SB, SK, AS, MR-S, MP, and CM. Manuscript writing: LV, JP, and DJ. Constructive review of manuscript: JJ, SB, AS, WV, MR-S, ZW, AM, MP, and CM. All authors contributed to the article and approved the submitted version.

## Funding

This manuscript results in part from collaboration and network activities promoted under the frame of the international network GENIEUR (Genes in Irritable Bowel Syndrome Research Network Europe), which has been funded by the COST program (BM1106, www.GENIEUR.eu) and is currently supported by the European Society of Neurogastroenterology and Motility (ESNM, www.ESNM.eu).

## Conflict of Interest

Part of the work of JP is financed by the Joint Programming Initiative A healthy diet for a healthy life (HDHL) Joint Action Intestinal Microbiomics (project number 50–52905–98–599). WV was partially supported by the SIAM Gravitation Grant 024.002.002 and Spinoza Award of the Netherlands Organization for Scientific Research. MR-S performed consultation services for Hemofarm AD, Serbia. ZW was supported to attend a scientific meeting by Will Pharma S.A. AM has received a ZonMw, The Netherlands Organization for Health Research and Development, health care efficiency grant to evaluate efficacy of peppermint oil in IBS, has received an unrestricted research grant from Will Pharma S.A., and received research funding from Allergan and Grünenthal on IBS topics. AM has given scientific advice to Bayer and Kyowa Kirin related to IBS and constipation, and received funding from Pentax Europe GmBH. Part of the work of CM is supported by the Instituto de Salud Carlos III, grant PI/17/00614 co-financed by the European Regional Development Fund (ERDF). Part of the work of DJ is financed by Grant Top Knowledge Institute (Well on Wheat), the Carbokinietics program as part of the NWO-CCC Partnership program and H2020 Nr. 848228/DISCOvERIE.

The remaining authors declare that the research was conducted in the absence of any commercial or financial relationships that could be construed as a potential conflict of interest.

## Supplementary Material

Supplementary Table 1A Inter-item correlations (Pearson correlations) between observed species of consecutive samples, for healthy subjects and IBS patients separately.

Supplementary Table 1B Inter-item correlations (Pearson correlations) between effective Shannon index of consecutive samples, for healthy subjects and IBS patients separately.

Supplementary Table 2A Results from linear mixed-effects models (with random intercept, fixed slopes, and scaled identity covariance structure). Regression coefficient indicates the direction and strength of the association between the predictor and dependent variable.

Supplementary Table 2B Results from linear mixed-effects models (with random intercept, fixed slopes, and scaled identity covariance structure). Regression coefficient indicates the direction and strength of the association between the predictor and dependent variable.

Supplementary Figure 1 Stool consistency for each sample and separately for all subjects (*i.e.* each color represents a subject), measured by fecal dry weight percentage **(A)** and Bristol Stool Scale **(B)**.

Supplementary Figure 2 Overview of the microbial composition of IBS and healthy subjects based on relative abundances on the phylum level.

Supplementary Figure 3 Relative abundances for healthy subjects *vs.* IBS patients, significance tested using Mann Whitney U test. **** p < 0.01, *NS*, Not significant. **(A)** Firmicutes, **(B)** Bacteroidetes, **(C)** Actinobacteria, **(D)** Verrucomicrobia, **(E)** Proteobacteria.

Supplementary Figure 4 **(A)** Generalized UniFrac PCoA plot per individual (different colors) and for healthy subjects *vs.* IBS patients (see figure annotations). **(B)** Average within-subject beta diversity (generalized UniFrac) for healthy subjects *vs.* IBS patients, significance tested using Mann Whitney U test, p = 0.80.

Supplementary Figure 5 Bray Curtis Dissimilarity for days 2-7 compared to day 1 for IBS patients **(A)** and healthy subjects **(B)**. Different colors representing individual subjects; black and dashed line representing the mean Bray Curtis Dissimilarity.
