# Supplementary material for: Does Day-to-Day Variability in Stool Consistency Link to the Fecal Microbiota Composition?
Source: Front Cell Infect Microbiol. 2021 Jul 20;11:639667. doi: 10.3389/fcimb.2021.639667 (PMC8386168; doi:10.3389/fcimb.2021.639667)
Supplement: Supplementary Table 1A — Inter-item correlations (Pearson correlations) between observed species of consecutive samples, for healthy subjects and IBS patients separately. [file DataSheet_1.zip › Supplementary Methods and Results.docx]

**SUPPORTING INFORMATION**

**Supplementary Methods**

DNA extraction

Approximately 125 mg of fecal matter was used for DNA extraction using the validated Repeated Bead Beating (RBB) method ^1, 2^ with the following modifications for automated DNA purification: feces was suspended in 1.0 ml of sterile ice-cold PBS, and 175 μl of fecal suspension was combined with 235 μl of RBB lysis buffer (500 mM NaCl, 50 mM Tris-HCl (pH 8.0), 50 mM EDTA, 4% SDS) in a bead beating tube from the Ambion Magmax™ Total Nucleic Acid Isolation Kit (Life Technologies, Carlsbad, CA, USA). After repeated bead beating, 200 μl of the supernatant was used for DNA extraction with the KingFisherTM Flex automated purification system (ThermoFisher Scientific, Waltham, MA, USA) using MagMAXTM Pathogen High Vol Duo program. DNA was quantified using Quanti-iT™ Pico Green dsDNA Assay (Invitrogen, San Diego, CA, USA).

High-throughput DNA sequencing

The microbiota composition was profiled by amplifying the hypervariable V4-region of the bacterial and archaeal 16S rRNA gene by PCR. The detailed description of the high-throughput amplicon sequencing protocol can be found in a previous publication ^3^. Briefly, forward V4F_515_19 (V4F_515_19: 5’-GTGCCAGCAMGCCGCGGTAA -3’) and 12-base Golay barcoded reverse primers V4R_806 (5’- GGACTACCAGGGTATCTAAT-3’) were used to amplify the V4-region.

Amplicons were purified using the QIAquick PCR Purification Kit (Qiagen, Barcelona, Spain), quantified using a NanoDrop ND-1000 Spectrophotometer (Nucliber) and then pooled in an equimolar concentration. The pooled amplicon library was subsequently subjected to sequencing using Illumina MiSeq technology at the technical support unit of the Autonomous University of Barcelona (UAB, Spain), following standard Illumina platform protocols.

Sequence data analysis

Raw sequences were loaded in a unique file into the QIIME software. Upon removal of low-quality reads with a Phred score smaller than 20, reads were demultiplexed. This resulted in 10.145.380 high quality reads (from 14.642.422 input sequences) for the 149 samples ranging from 36.107 to 93.479 reads per sample. Next, sequences were clustered into Operational Taxonomic Units (OTUs) with 97% of similarity using the USEARCH algorithm ^4^. Chimeric sequences were removed with UCHIME ^5^ and OTUs corresponding to singletons were eliminated.

To reduce the amount of data, a representative sequence from each OTU was picked and the resulting sequences were aligned against a GreenGenes template alignment using PyNAST as recommended by Navas-Molina *et al.* ^6^. Using the Basic Local Alignment Search Tool (BLAST) and a database that combines GreenGenes (gg_13_8 release) and PATRIC (Pathosystems Resource Integration Center) databases, taxonomy was assigned to each OTU. The OTU table, containing the taxonomical assignment per OTU and their abundances in each sample, was built and at this point, a total of 8.054.504 reads for the 149 samples ranging from 29.926 to 73.203 reads per sample, remained for downstream analyses. Data were normalized using a rarefaction technique, by randomly selecting 29.926 sequences (minimum number of reads in a sample) in each sample, in order to obtain the same number of reads per sample.

Using the FastTree program, the phylogenic tree was created, followed by computing Chao1 and Shannon indexes as measures of alpha diversity. Furthermore, (un)weighted and generalized UniFrac metrics, and Bray Curtis index were computed to evaluate between-sample diversity (beta diversity). Sequence data have been deposited in the NCBI database with the following access number: PRJNA682378.

**Supplementary Results**

Association between microbiota and GI symptoms

Within the IBS population, linear mixed-effects models showed that the association between the interaction term “abdominal pain*time” and Chao1 index was not significant (B: -0.034, SE: 0.203, p=0.869). Furthermore, no significant association was found between abdominal pain and Chao1 index (B: 0.473, SE: 0.839, p=0.574), after removal of the two-way interaction term from the model (Table 3). In models assessing abdominal bloating as an independent variable (including both the interaction “abdominal bloating*time” and abdominal bloating as fixed effects), also no significant association between abdominal bloating and fecal microbial richness was found (Table 3). Similar results were found for observed species and the effective Shannon index as dependent variables (Supplementary Table 2A and 2B).

Moreover, redundancy analysis again showed clear clustering per individual, with no significant association between abdominal pain and microbiota composition (p=0.545), nor between abdominal bloating and microbiota composition (p=0.855) (Figure 3). Since these redundancy analyses showed no significant association between abdominal symptoms and microbial composition, no subsequent analyses (*i.e.* linear mixed-effects models) on specific associations between different taxa and GI symptoms were performed.

**REFERENCES**

1. Salonen A, Nikkila J, Jalanka-Tuovinen J, Immonen O, Rajilic-Stojanovic M, Kekkonen RA*, et al.* Comparative analysis of fecal DNA extraction methods with phylogenetic microarray: effective recovery of bacterial and archaeal DNA using mechanical cell lysis. J Microbiol Methods. 2010;81(2):127-34.

2. J. D, Ehrlich SD, Levenez F, Pelletier E, Alberti A, Bertrand L*, et al.* IHMS_SOP 06 V1: Standard operating procedure for fecal samples DNA extraction, Protocol Q. . International Human Microbiome Standards. 2015.

3. Pascal V, Pozuelo M, Borruel N, Casellas F, Campos D, Santiago A*, et al.* A microbial signature for Crohn's disease. Gut. 2017;66(5):813-22.

4. Edgar RC. Search and clustering orders of magnitude faster than BLAST. Bioinformatics. 2010;26(19):2460-1.

5. Edgar RC, Haas BJ, Clemente JC, Quince C, Knight R. UCHIME improves sensitivity and speed of chimera detection. Bioinformatics. 2011;27(16):2194-200.

6. Navas-Molina JA, Peralta-Sanchez JM, Gonzalez A, McMurdie PJ, Vazquez-Baeza Y, Xu Z*, et al.* Advancing our understanding of the human microbiome using QIIME. Methods Enzymol. 2013;531:371-444.

**SUPPLEMENTARY TABLES**

**Supplementary Table 1A**: Inter-item correlations (Pearson correlations) between observed species of consecutive samples, for healthy subjects and IBS patients separately.

| *Observed species* | HEALTHY SUBJECTS | | | | IBS PATIENTS | | | | |
| --- | --- | --- | --- | --- | --- | --- | --- | --- | --- |
|  | *Intraclass Correlations Coefficient = 0.935* | | | | *Intraclass Correlations Coefficient = 0.879* | | | | |
|  | **Sample 1** | **Sample 2** | **Sample 3** | **Sample 4** | **Sample 1** | **Sample 2** | **Sample 3** | **Sample 4** |  |
| **Sample 2** | 0.965 |  |  |  | 0.886 |  | | | |
| **Sample 3** | 0.868 | 0.927 |  |  | 0.849 | 0.891 |  | | |
| **Sample 4** | 0.971 | 0.943 | 0.904 |  | 0.818 | 0.908 | 0.946 |  |  |
| **Sample 5** | 0.946 | 0.990 | 0.929 | 0.912 | 0.907 | 0.877 | 0.932 | 0.917 |  |

**Supplementary Table 1B**: Inter-item correlations (Pearson correlations) between effective Shannon index of consecutive samples, for healthy subjects and IBS patients separately.

| *Effective Shannon index* | HEALTHY SUBJECTS | | | | IBS PATIENTS | | | |  |
| --- | --- | --- | --- | --- | --- | --- | --- | --- | --- |
|  | *Intraclass Correlations Coefficient = 0.612* | | | | *Intraclass Correlations Coefficient = 0.519* | | | |  |
|  | **Sample 1** | **Sample 2** | **Sample 3** | **Sample 4** | **Sample 1** | **Sample 2** | **Sample 3** | **Sample 4** | |
| **Sample 2** | 0.633 |  |  |  | 0.020 |  | | | |
| **Sample 3** | 0.598 | 0.913 |  |  | 0.312 | 0.872 |  | | |
| **Sample 4** | 0.780 | 0.516 | 0.712 |  | 0.056 | 0.746 | 0.611 |  | |
| **Sample 5** | 0.432 | 0.831 | 0.613 | 0.173 | 0.537 | 0.616 | 0.866 | 0.386 | |

**Supplementary Table 2A**: Results from linear mixed-effects models (with random intercept, fixed slopes, and scaled identity covariance structure). Regression coefficient indicates the direction and strength of the association between the predictor and dependent variable.

| **Dependent variable** | **Predictor** | **Regression coefficient**  **[95%-CI]** | **SE** | **p-value** |
| --- | --- | --- | --- | --- |
| *Observed species* | Fecal dry weight*time | 0.028  [-0.095; 0.151] | 0.062 | 0.651 |
|  | Fecal dry weight^1^ | 1.066  [0.725; 1.407] | 0.172 | <0.001 |
|  | BSS*time | -0.050  [-0.786; 0.687] | 0.372 | 0.894 |
|  | BSS^1^ | -2.190  [-4.116; -0.264] | 0.973 | 0.026 |
|  | Abdominal pain*time | -0.074  [-0.420; 0.273] | 0.175 | 0.674 |
|  | Abdominal pain^1^ | 0.626  [-0.806; 2.058] | 0.723 | 0.388 |
|  | Abdominal bloating*time | 0.418  [-0.005; 0.840] | 0.213 | 0.053 |
|  | Abdominal bloating^1^ | 0.507  [-1.004; 2.019] | 0.763 | 0.508 |

^1^ Insignificant interaction terms, respectively, “fecal dry weight*time”, “BSS*time” “abdominal pain*time”, and “abdominal bloating*time” were removed from the models. BSS: Bristol Stool Scale. SE: standard error.

**Supplementary Table 2B**: Results from linear mixed-effects models (with random intercept, fixed slopes, and scaled identity covariance structure). Regression coefficient indicates the direction and strength of the association between the predictor and dependent variable.

| **Dependent variable** | **Predictor** | **Regression coefficient**  **[95%-CI]** | **SE** | **p-value** |
| --- | --- | --- | --- | --- |
| *Effective Shannon index* | Fecal dry weight *time | -0.289  [-0.839; 0.261] | 0.278 | 0.300 |
|  | Fecal dry weight^1^ | 3.159  [1.720; 4.598] | 0.727 | <0.001 |
|  | BSS*time | -0.2085  [-5.324; 1.153] | 1.635 | 0.205 |
|  | BSS^1^ | -2.921  [-11.143; 5.302] | 4.152 | 0.483 |
|  | Abdominal pain*time | -0.537  [-1.961; 0.886] | 0.719 | 0.456 |
|  | Abdominal pain^1^ | -4.283  [-9.694; 1.128] | 2.733 | 0.120 |
|  | Abdominal bloating*time | 0.713  [-1.062; 2.488] | 0.896 | 0.428 |
|  | Abdominal bloating^1^ | -2.459  [-8.280; 3.364] | 2.940 | 0.405 |

^1^ Insignificant interaction terms, respectively, “fecal dry weight*time”, “BSS*time” “abdominal pain*time”, and “abdominal bloating*time” were removed from the models. BSS: Bristol Stool Scale. SE: standard error.
