# Supplementary figures and images for: Does Day-to-Day Variability in Stool Consistency Link to the Fecal Microbiota Composition?
Source: Front Cell Infect Microbiol. 2021 Jul 20;11:639667. doi: 10.3389/fcimb.2021.639667 (PMC8386168; doi:10.3389/fcimb.2021.639667)

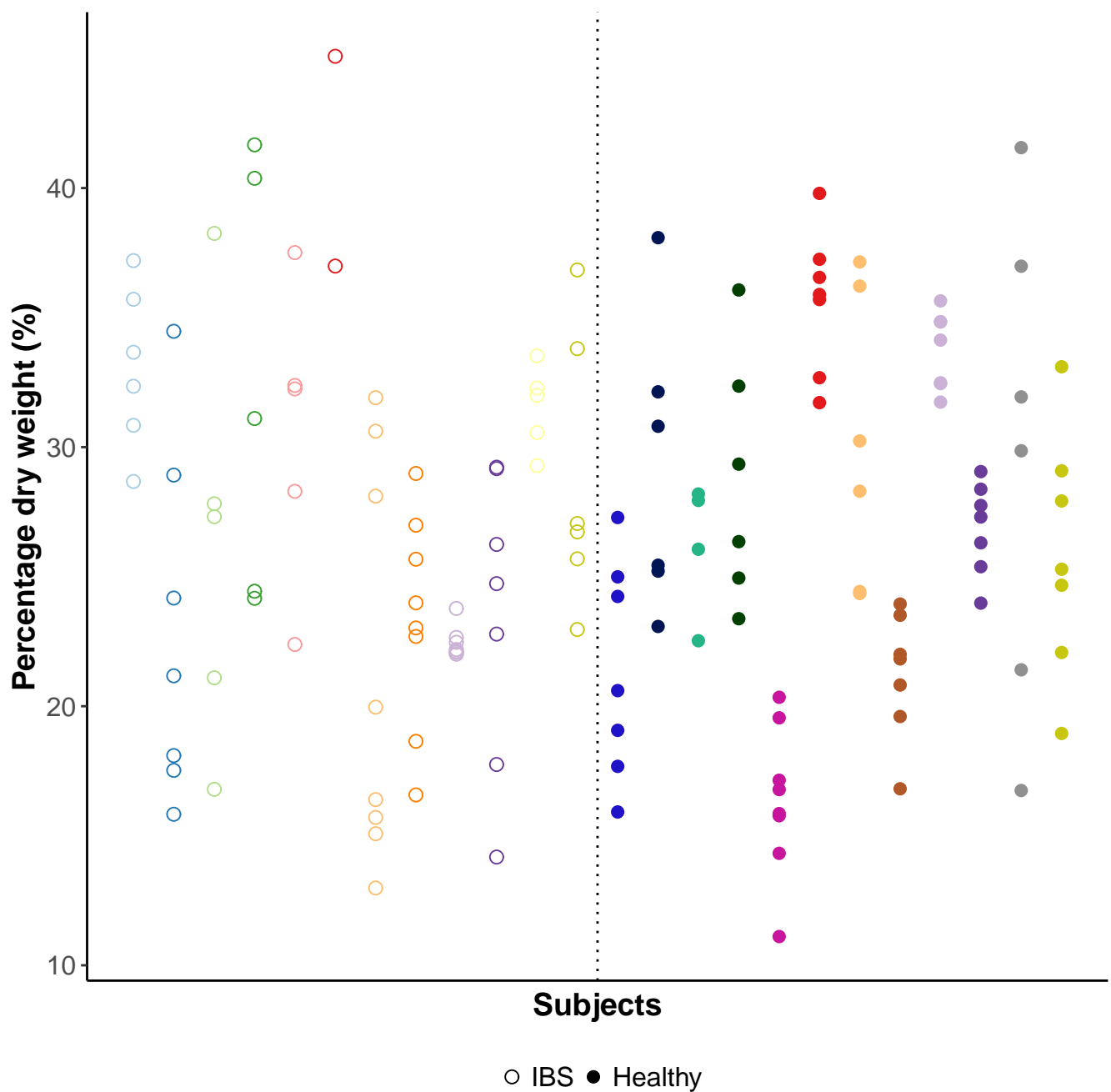

Supplement: Supplementary Table 1A — Inter-item correlations (Pearson correlations) between observed species of consecutive samples, for healthy subjects and IBS patients separately. [file DataSheet_1.zip › Supplementary Figure 1A.pdf]

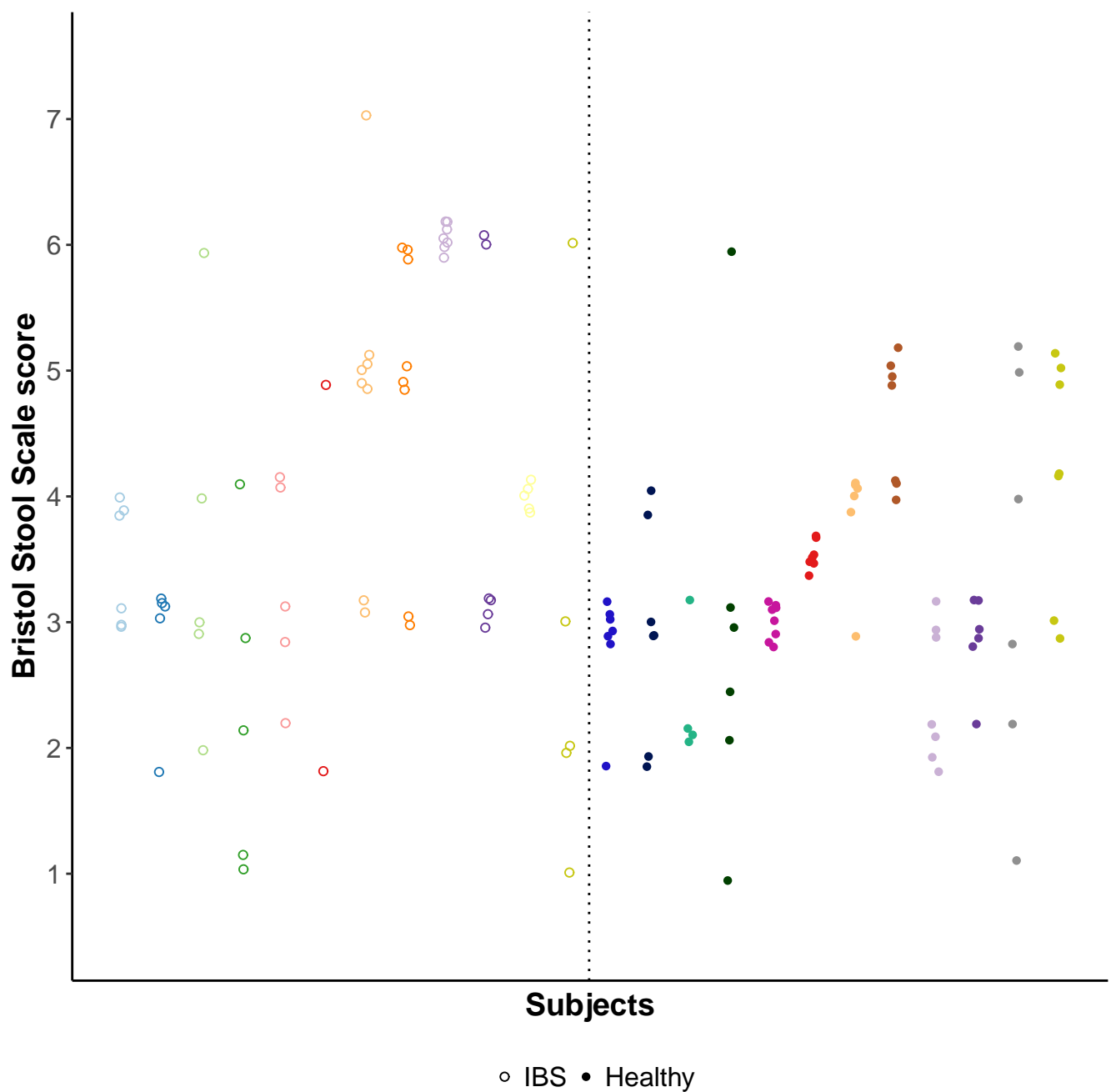

Supplement: Supplementary Table 1A — Inter-item correlations (Pearson correlations) between observed species of consecutive samples, for healthy subjects and IBS patients separately. [file DataSheet_1.zip › Supplementary Figure 1B.pdf]

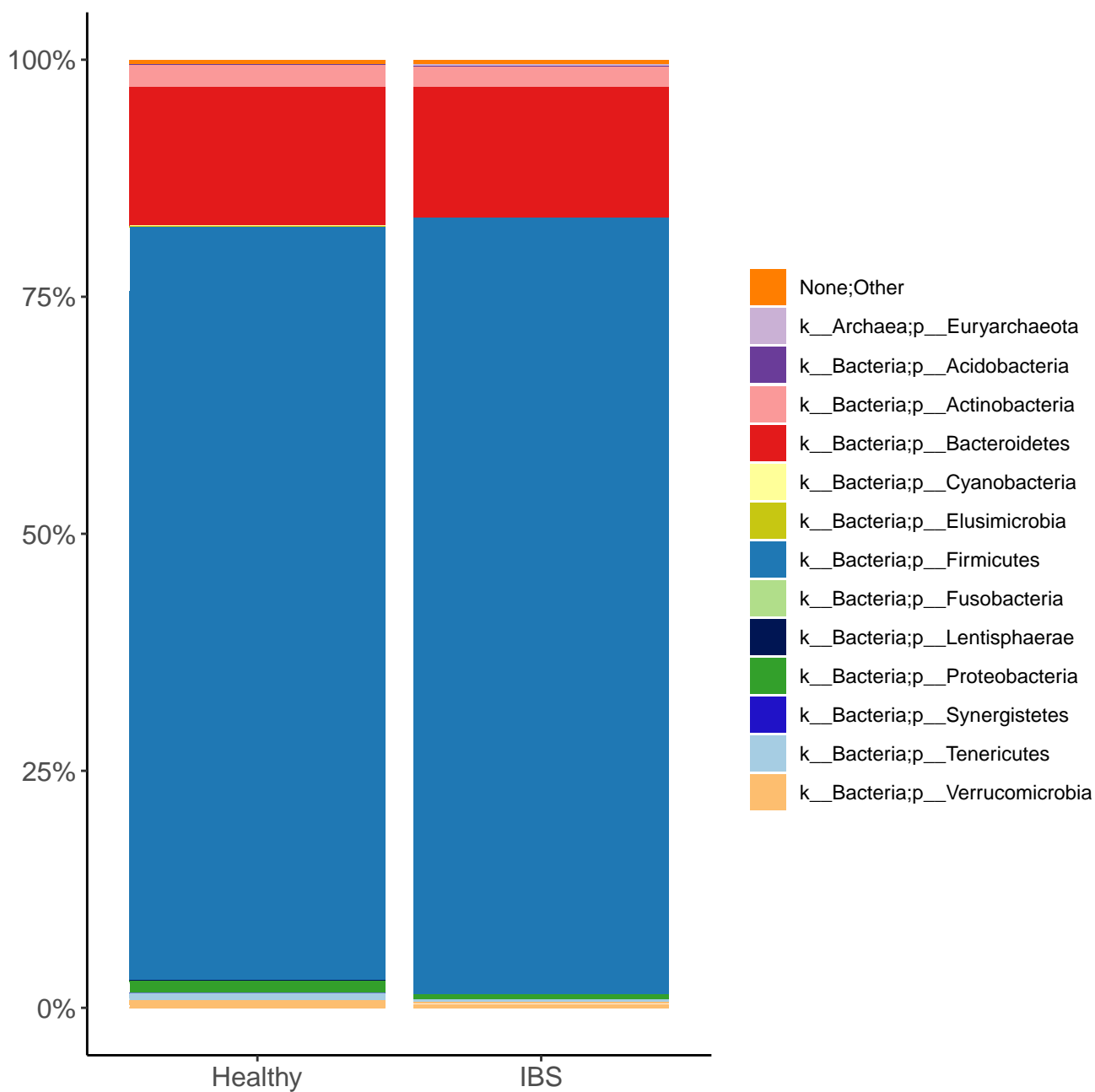

Supplement: Supplementary Table 1A — Inter-item correlations (Pearson correlations) between observed species of consecutive samples, for healthy subjects and IBS patients separately. [file DataSheet_1.zip › Supplementary Figure 2.pdf]

# Firmicutes

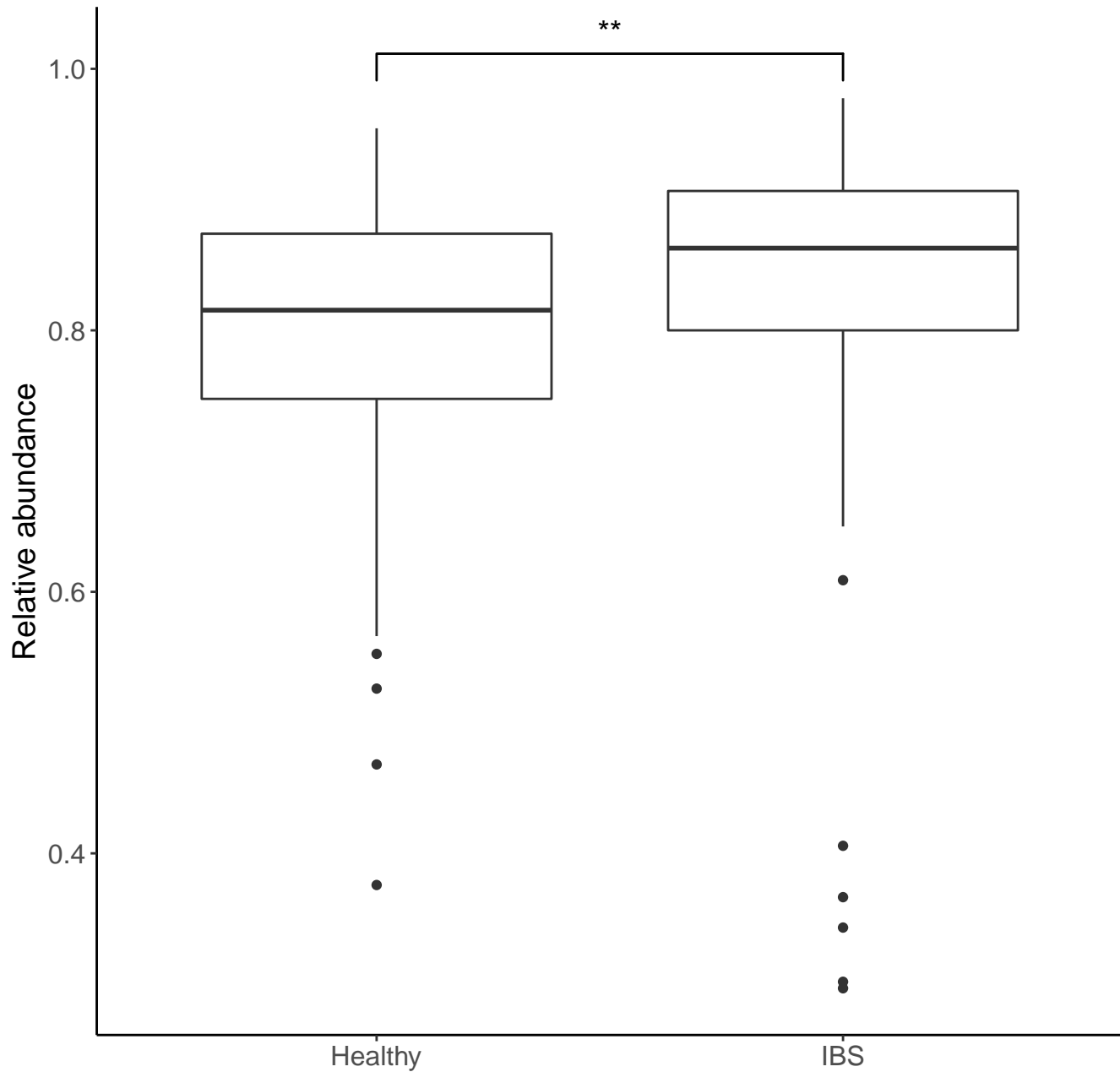

Supplement: Supplementary Table 1A — Inter-item correlations (Pearson correlations) between observed species of consecutive samples, for healthy subjects and IBS patients separately. [file DataSheet_1.zip › Supplementary Figure 3A.pdf]

# Bacteroidetes

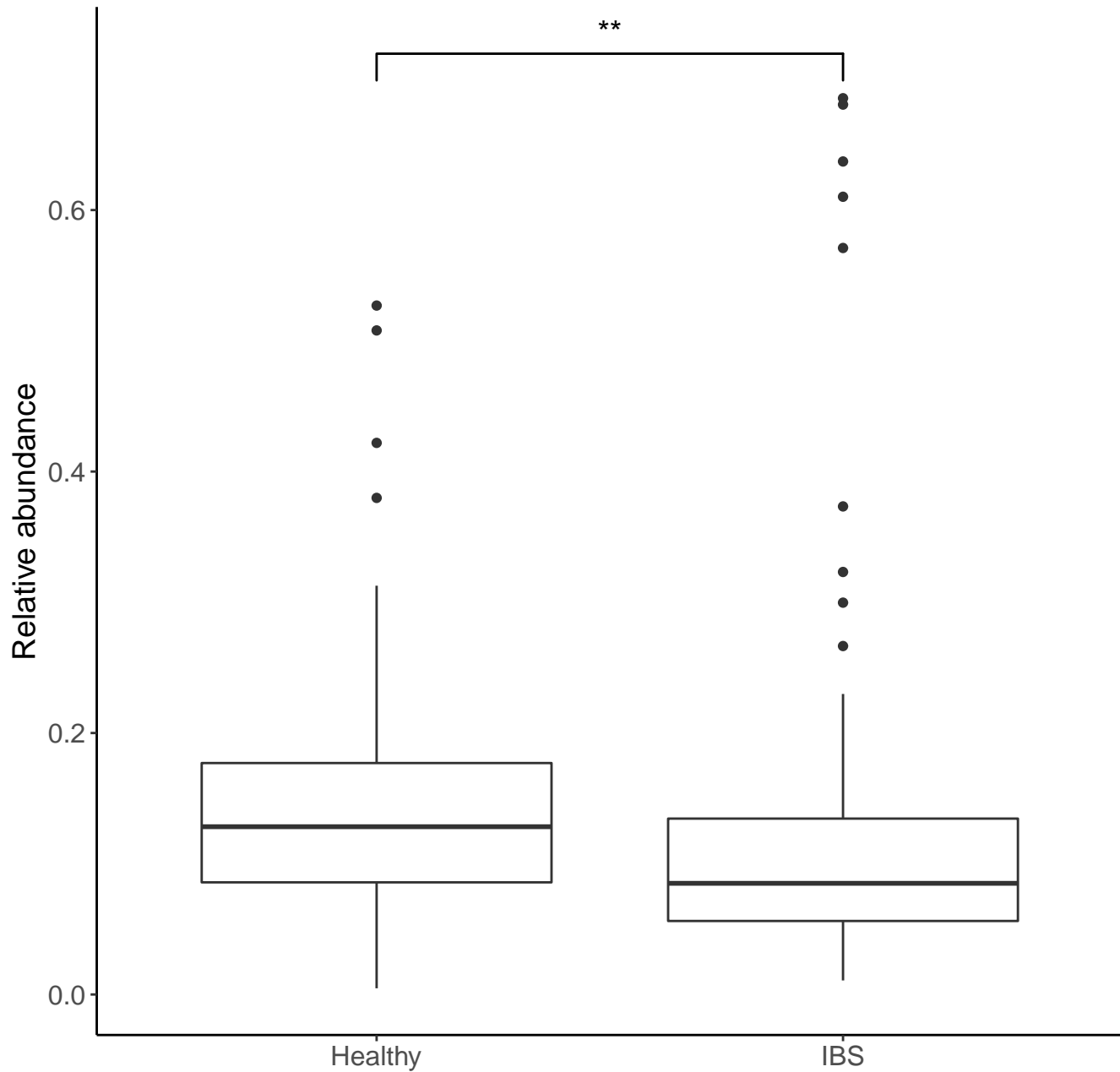

Supplement: Supplementary Table 1A — Inter-item correlations (Pearson correlations) between observed species of consecutive samples, for healthy subjects and IBS patients separately. [file DataSheet_1.zip › Supplementary Figure 3B.pdf]

# Actinobacteria

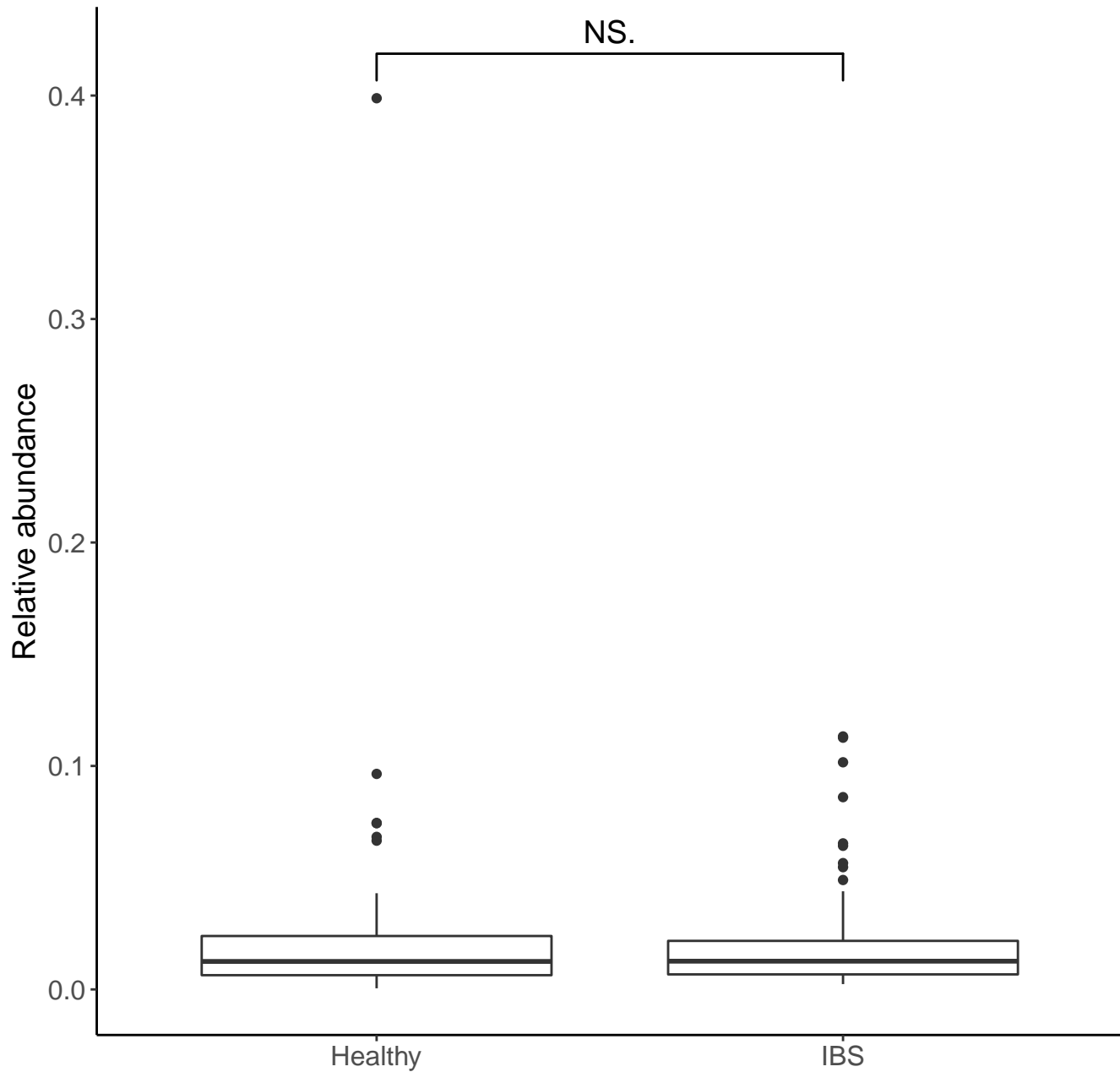

Supplement: Supplementary Table 1A — Inter-item correlations (Pearson correlations) between observed species of consecutive samples, for healthy subjects and IBS patients separately. [file DataSheet_1.zip › Supplementary Figure 3C.pdf]

# Verrucomicrobia

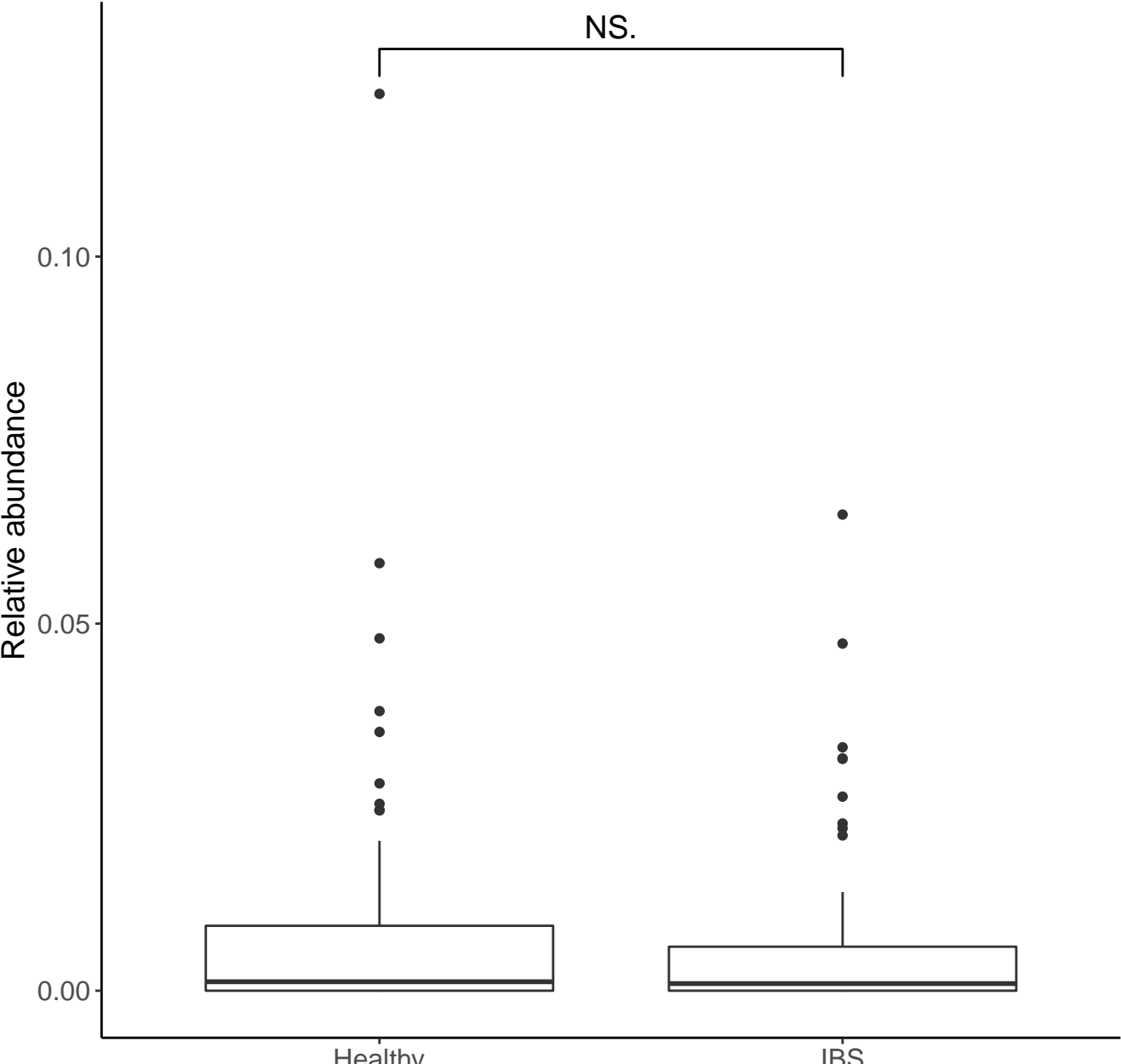

Supplement: Supplementary Table 1A — Inter-item correlations (Pearson correlations) between observed species of consecutive samples, for healthy subjects and IBS patients separately. [file DataSheet_1.zip › Supplementary Figure 3D.pdf]

# Proteobacteria

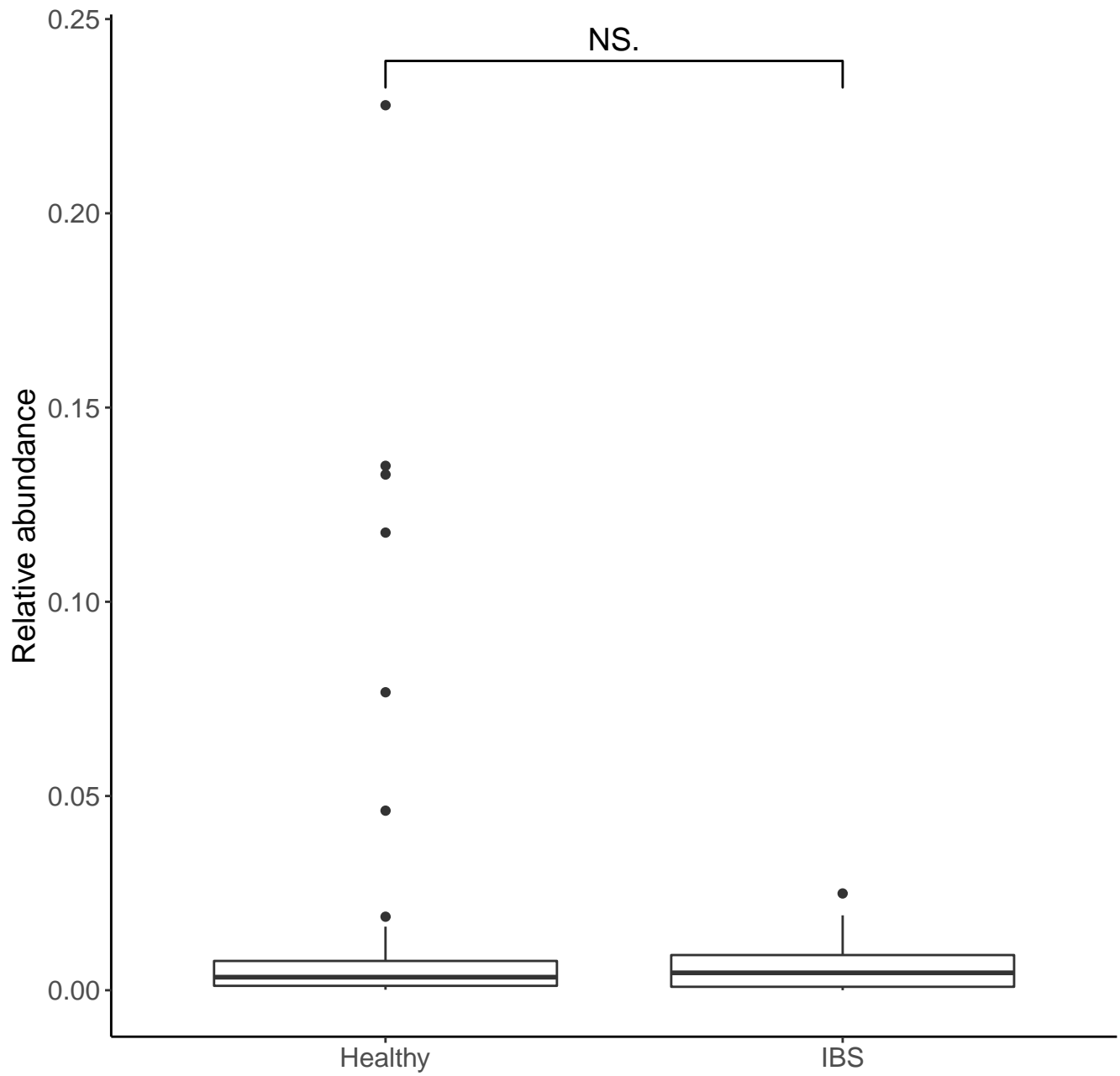

Supplement: Supplementary Table 1A — Inter-item correlations (Pearson correlations) between observed species of consecutive samples, for healthy subjects and IBS patients separately. [file DataSheet_1.zip › Supplementary Figure 3E.pdf]

# Generalized UniFrac Distance

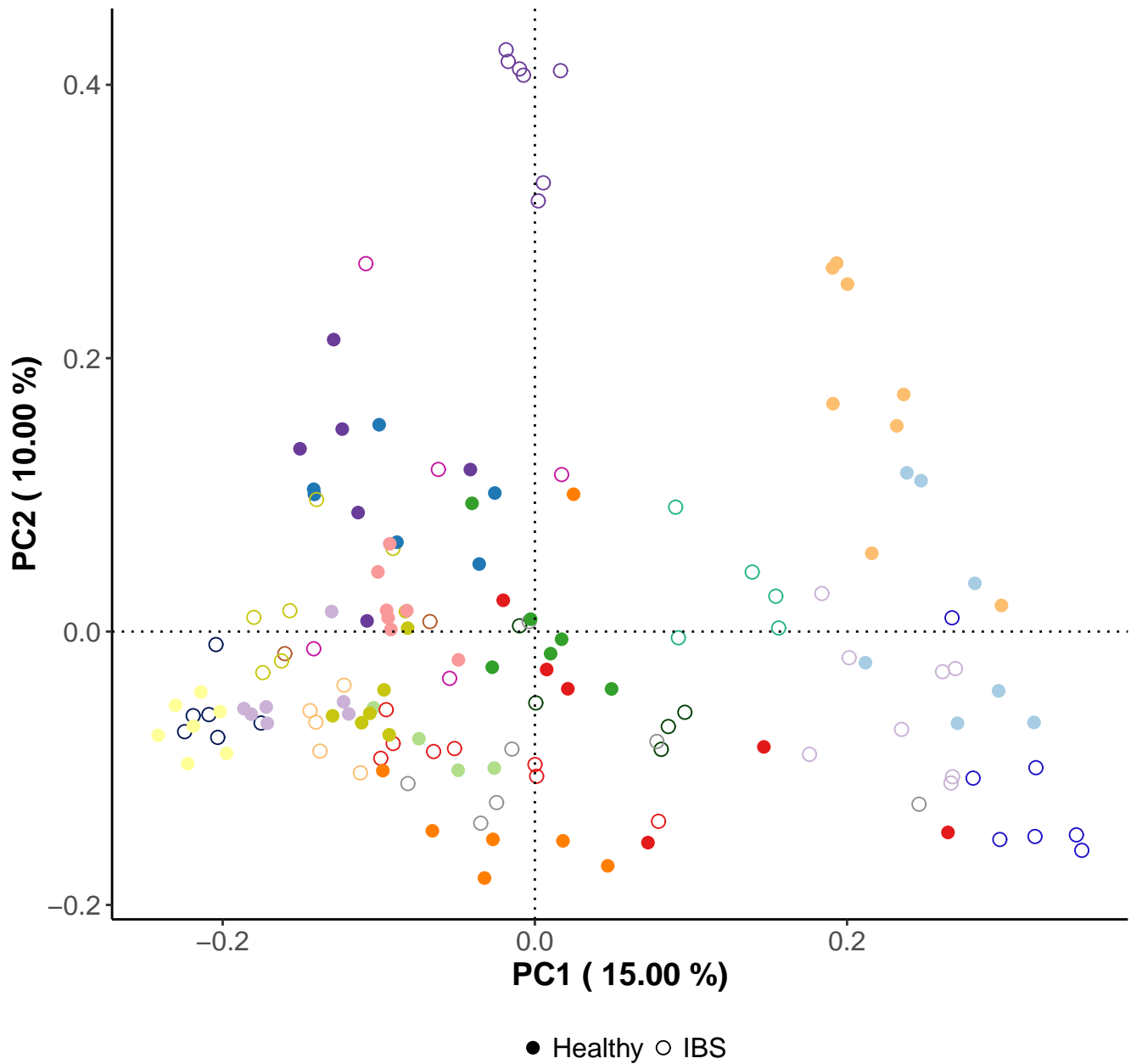

Supplement: Supplementary Table 1A — Inter-item correlations (Pearson correlations) between observed species of consecutive samples, for healthy subjects and IBS patients separately. [file DataSheet_1.zip › Supplementary Figure 4A.pdf]

# Generalized Unifrac Distance

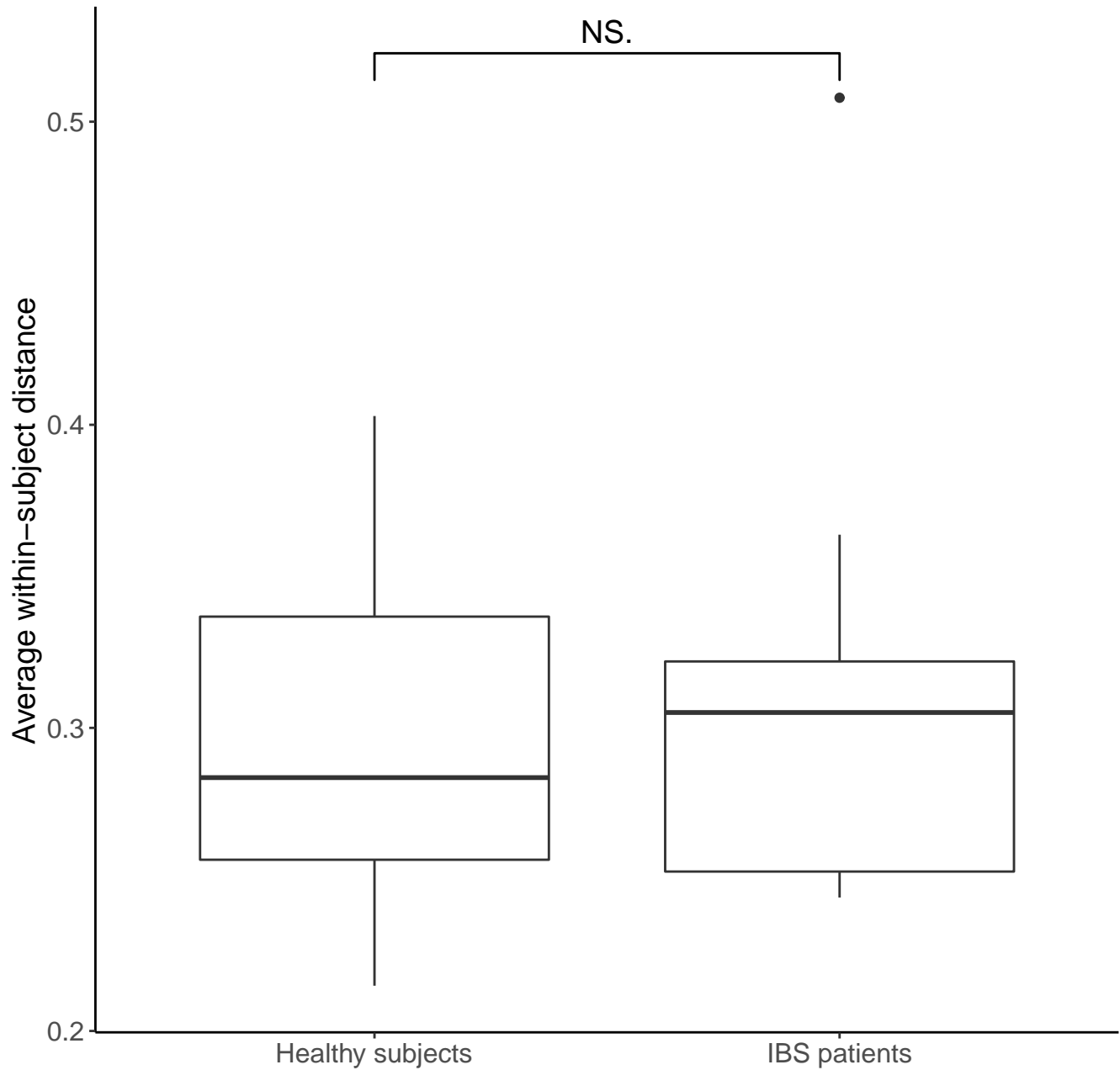

Supplement: Supplementary Table 1A — Inter-item correlations (Pearson correlations) between observed species of consecutive samples, for healthy subjects and IBS patients separately. [file DataSheet_1.zip › Supplementary Figure 4B.pdf]

# Bray Curtis Dissimilarity – IBS patients

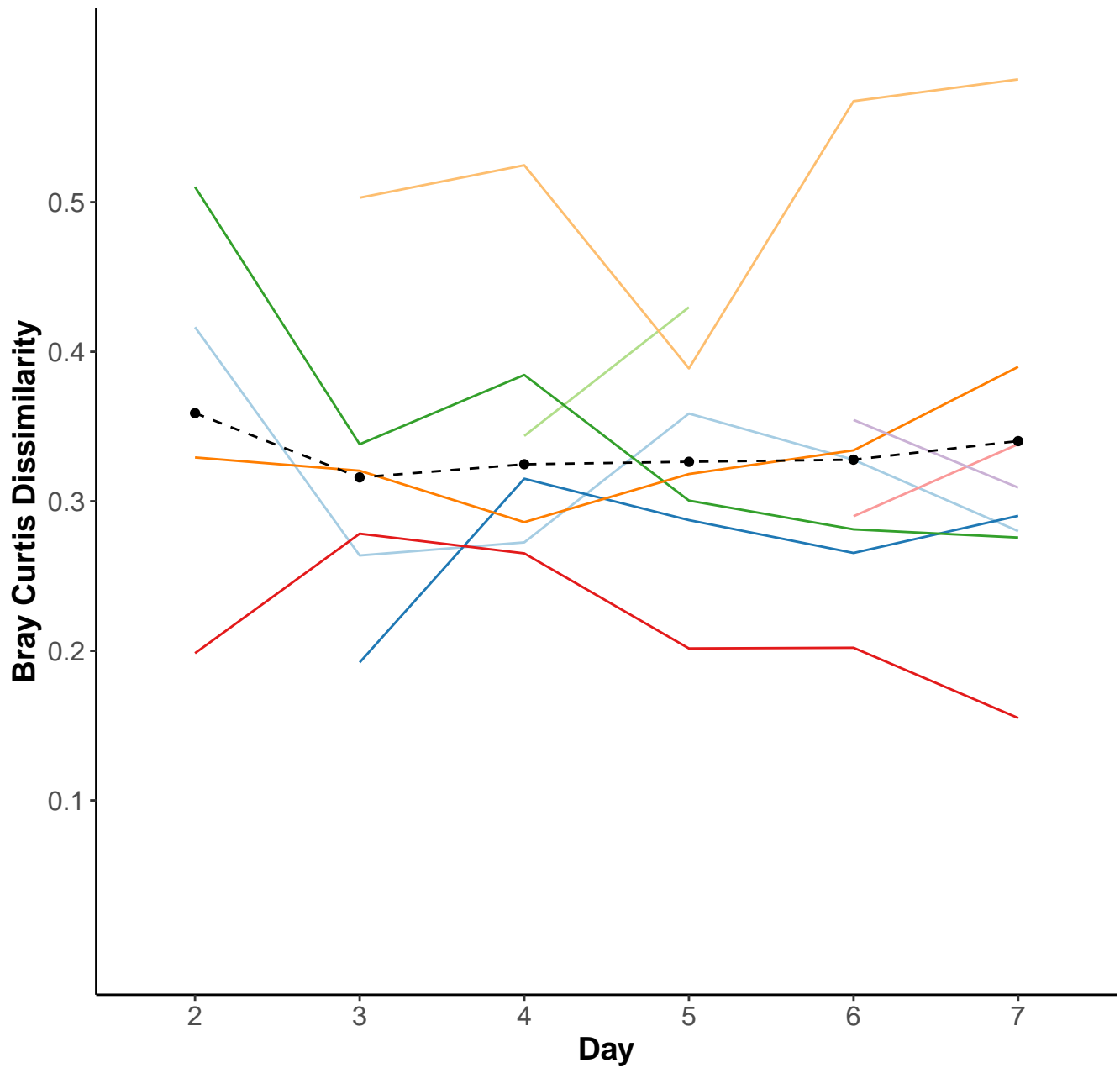

Supplement: Supplementary Table 1A — Inter-item correlations (Pearson correlations) between observed species of consecutive samples, for healthy subjects and IBS patients separately. [file DataSheet_1.zip › Supplementary Figure 5A.pdf]

# Bray Curtis Dissimilarity – Healthy subjects

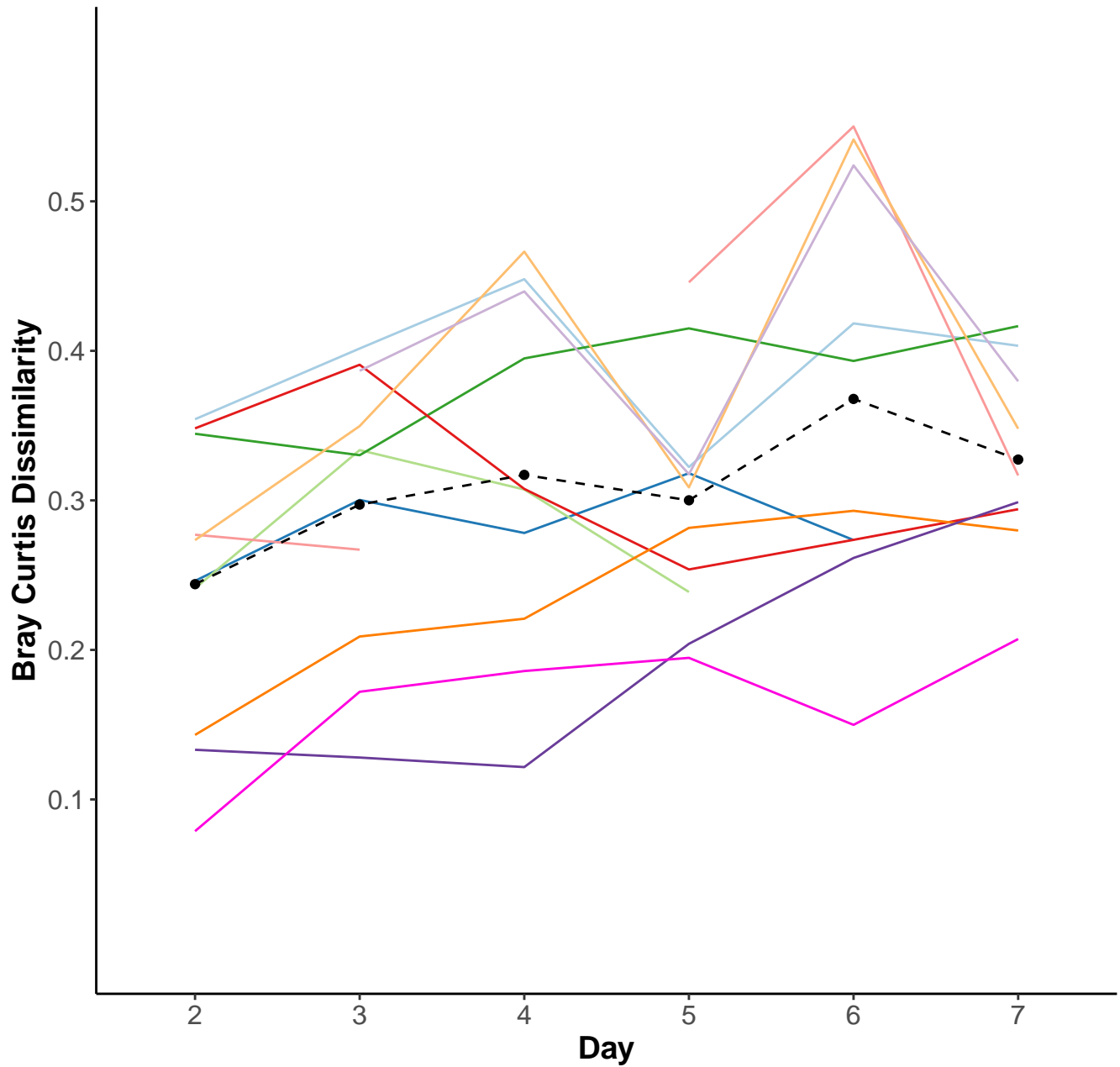

Supplement: Supplementary Table 1A — Inter-item correlations (Pearson correlations) between observed species of consecutive samples, for healthy subjects and IBS patients separately. [file DataSheet_1.zip › Supplementary Figure 5B.pdf]
